# Mesoporous Silica Nanoparticles Loaded with Cisplatin and Phthalocyanine for Combination Chemotherapy and Photodynamic Therapy *in vitro*

**DOI:** 10.3390/nano5042302

**Published:** 2015-12-16

**Authors:** Juan L. Vivero-Escoto, Maram Elnagheeb

**Affiliations:** 1 Department of Chemistry, University of North Carolina at Charlotte, Charlotte, NC 28223, USA; E-Mail: melnagheeb@gmail.com; 2 Center for Biomedical Engineering and Science, University of North Carolina at Charlotte, Charlotte, NC 28223, USA

**Keywords:** mesoporous silica nanoparticles, photodynamic therapy, chemotherapy, cisplatin, phototoxicity, phthalocyanine, fluorescent imaging

## Abstract

Mesoporous silica nanoparticles (MSNs) have been synthesized and loaded with both aluminum chloride phthalocyanine (AlClPc) and cisplatin as combinatorial therapeutics for treating cancer. The structural and photophysical properties of the MSN materials were characterized by different spectroscopic and microscopic techniques. Intracellular uptake and cytotoxicity were evaluated in human cervical cancer (HeLa) cells by confocal laser scanning microscopy (CLSM) and 3-(4,5-dimethylthiazol-2-yl)-5-(3-carboxymethoxyphenyl)-2-(4-sulfophenyl)-2H-tetrazolium (MTS) assays, respectively. The CLSM experiments showed that the MSN materials can be readily internalized in HeLa cells. The cytotoxic experiments demonstrated that, after light exposure, the combination of both AlClPc and cisplatin compounds in the same MSN platform potentiate the toxic effect against HeLa cells in comparison to the control AlClPc-MSN and cisplatin-MSN materials. These results show the potential of using MSN platforms as nanocarriers for combination photodynamic and chemotherapies to treat cancer.

## 1. Introduction

Under illumination of light of a specific wavelength, photoactive molecules, called photosensitizers (PSs), will generate singlet oxygen species (^1^O_2_) or reactive oxygen species (ROS), both of which are toxic to cancer cells [1,2,3,4]. The major advantage of this therapeutic approach, called photodynamic therapy (PDT), is that it is non-invasive and, therefore, more intrinsically safe compared to traditional cancer therapy modalities, such as surgery, radiotherapy, and chemotherapy. In the absence of illumination with suitable wavelengths of light, PS molecules cannot be activated and cannot generate toxic products and are, thus, safe to cells and tissues. Various PSs, such as Photofrin®, methylene blue, 5-aminolevulinic acid and chlorin e6, have been widely used both in fundamental research and in clinical applications [5,6,7]. Ideal characteristics of PS molecules include high toxicity only in the presence of light, selectivity and specificity for tumors, high quantum yields of singlet oxygen production, and absorption wavelengths between 600 and 800 nm [8]. An additional benefit is that fluorescence imaging can often be used to guide PDT because many PSs are also fluorescent [9].

Traditionally, PS agents, such as porphyrins and phthalocyanines, have dominated the field [6,10]. However, these PS agents have multiple drawbacks, such as low water solubility, poor light absorption, cutaneous photosensitivity, and low selectivity for targeted tissues. Several nanoparticle-based approaches have been developed to deliver PS molecules with the purpose of improving the efficacy of PDT [11,12,13,14,15]. Organic nanoparticles such as liposomes, micelles, polysaccharides, dendrimers, and polymers have been extensively used in the delivery of PSs [14,16,17]. In addition, different inorganic materials with unique and interesting properties such as silica-based nanostructures, metallic nanoparticles, magnetic nanoparticles, quantum dots, as well as carbon nanomaterials, have also been explored for delivering PS agents [14,18]. Silica-based nanoparticles, like solid silica, polysilsesquioxanes, and mesoporous silica nanoparticles, present the unique advantages of being nontoxic, having tunable surfaces, displaying chemical inertness and being optically transparent [18,19,20,21,22,23]. In particular, mesoporous silica nanoparticles (MSNs) have recently attracted a great deal of attention as carriers of PS molecules. MSNs show outstanding properties, such as high surface areas, large pore volumes, tunable pore diameters, easy modification, chemical stability and good biocompatibility [24]. The use of MSNs as PS nanocarriers has been extensively explored over the past decade. Mou, Bein, and others have reported on the incorporation of PpIX, Pd-mesotetra(4-carboxyphenyl) porphyrin, aluminium phthalocyaninedisulfonate, and zinc(II) phthalocyanine within MSNs [25,26,27,28,29,30,31,32,33,34].

Combined treatments are commonly used to enhance the therapeutic outcome of anticancer drugs. Several studies have demonstrated the effectiveness of PDT combined with chemotherapy [35,36]. MSN platforms have recently been used for the effective transport and delivery of both photosensitizers and anticancer drugs. Photosensitizers such as porphyrins, chlorines, and fullerenes have been successfully incorporated in the framework of MSN material together with anticancer drugs such as doxorubicin to obtain enhanced therapeutic efficacy *in vitro* and *in vivo* [33,37,38,39]. Cisplatin is an alkylating agent clinically used to treat a wide variety of cancer types, including ovarian, lung, breast, and cervical cancers. However, cisplatin shows poor therapeutic efficacy against some of those cancer cell lines [40,41]. Herein, we use MSNs to carry a combined payload of both aluminum chloride phthalocyanine (AlClPc) and cisplatin as a combinatorial therapeutic strategy to treat cancer. MSNs were synthesized via a surfactant-templated approach and further loaded with AlClPc and/or cisplatin compounds. The amounts of AlClPc and cisplatin loaded into MSNs were quantified by UV-VIS and atomic absorption spectroscopy, respectively. The structural and photophysical properties of these MSN materials were characterized with a wide variety of spectroscopic and microscopic techniques. The *in vitro* internalization, and cyto- and phototoxicity tests were performed by confocal laser scanning microscopy (CLSM) and MTS assays using human cervical cancer (HeLa) cells.

## 2. Results and Discussion

### 2.1. Synthesis and Characterization of the Structural Properties of MSNs, AlClPc–MSNs, Cisplatin-MSNs, and AlClPc/Cisplatin–MSNs

**Figure 1:**
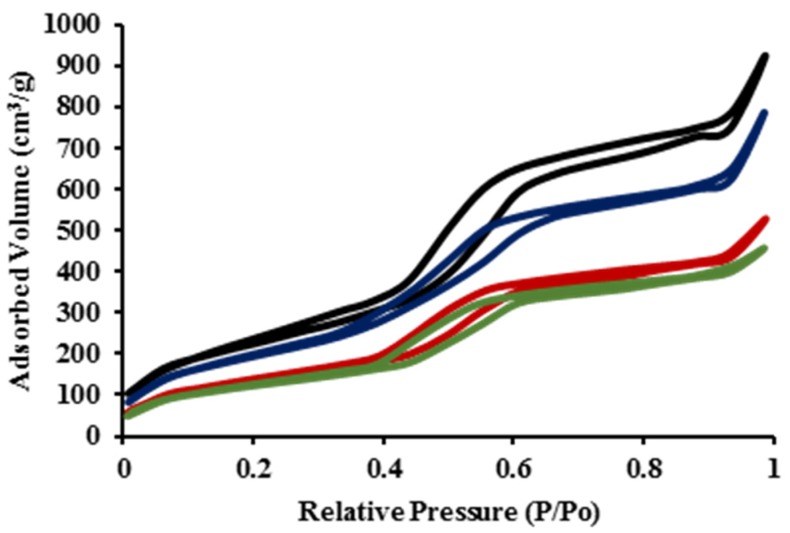
Nitrogen sorption isotherms of MSNs (**black**), AlClPc-MSNs (**blue**), cisplatin-MSNs (**red**), and AlClPc/cisplatin-MSNs (**green**).

**Figure 2:**
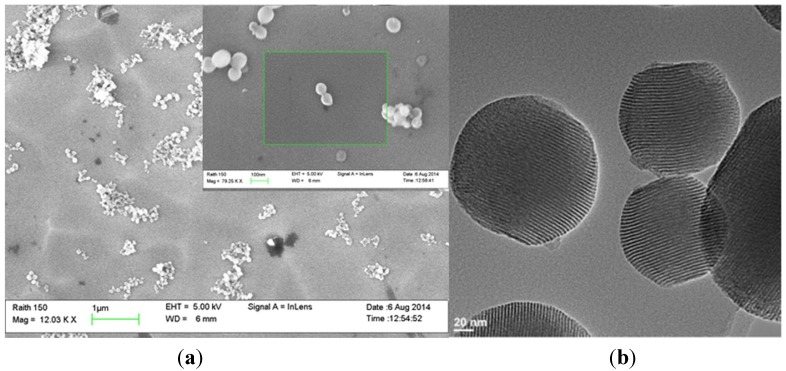
(**a**) Scanning electron microscopy (SEM) micrograph of MSNs (Scale bar: 1 µm). Inset: close up of MSNs (scale bar: 100 nm). (**b**) Transmission electron microscopy (TEM) image of MSNs (scale bar: 20 nm).

MSNs were synthesized through a surfactant-templated approach using cetyltrimethylammonium bromide (CTAB) as the surfactant [42]. The as-made MSN material was washed several times in an acidic solution of methanol (1.0 M) to remove the surfactant from the MSN framework, as described in the experimental section. AlClPc and cisplatin molecules were physically loaded into MSN particles after stirring the compounds for 24 h in dimethylsulfoxide (DMSO) solution in the presence of MSNs. The structural properties of MSNs, AlClPc–MSNs, cisplatin–MSNs, and AlClPc/cisplatin-MSNs were characterized by dynamic light scattering (DLS), ζ-potential, N_2_ sorption isotherms (Figure 1), thermogravimetric analysis (TGA), and scanning and transmission electron microscopy (SEM and TEM). DLS results show that the unmodified MSNs have an average hydrodynamic diameter of 96.5 ± 10.5 nm (Table 1 and Figure S1a) in phosphate buffer solution (PBS, 1 mM, pH 7.4; MSNs = 0.1 mg/mL), which is similar to the average particle diameter observed in SEM and TEM (109.7 ± 13.3 nm, Figure 2). However, when the hydrodynamic diameter of bare MSNs is determined in cell culture media (10 v % fetal bovine serum, FBS) the size is almost twice as large than the one measured in PBS (Table 1 and Figure S1e). This is mainly due to culture medium ionic effects [43]. The surface of the MSNs is negatively charged due to the presence of deprotonated silanols (siloxides), as indicated by the ζ-potential (−41.9 ± 2.0 mV). BET (Brunauer-Emmett-Teller) and BJH (Barrett-Joyner-Halenda) methods were used to calculate the surface area and plot the pore size distribution of the MSN materials fabricated in this work (Table 1 and Figure S2). As expected, bare MSNs have high surface area (819.7 m^2^/g), large pore size (5.3 nm), and volume (1.57 cm^3^/g). After the loading of the photosensitizer (AlClPc) and/or the anticancer drug (cisplatin), the hydrodynamic diameter of the MSN materials moderately increased in PBS; nevertheless, similar to bare MSNs, the hydrodynamic diameter in cell culture media also increased almost twice the original size (Table 1 and Figure S1b–d,f–h). The surface charges of the AlClPc-, cisplatin-, and AlClPc/cisplatin-MSNs remained negative, with some slight variations (−45.2 ± 3.2, −32.1 ± 1.1, and −23.1 ± 2.4 mV, respectively). A reduction in the surface area was observed, along with decreased pore sizes and volumes, which indicate loading of AlClPc and/or cisplatin molecules (Figure 1 and Table 1). TGA data corroborate the loading of AlClPc molecules into the framework of the MSNs. An increase of 7.4 wt % in weight loss, compared with the unmodified MSNs, was observed for AlClPc-MSNs, which corresponds to 144 µmol of AlClPc per gram of MSN material. UV-VIS spectroscopy as measured at 680 nm, the wavelength of maximum absorbance of the AlClPc molecule, confirmed a similar value of 122.8 ± 20.5 µmol. The amount of cisplatin loaded into the MSNs was 416.0 ± 106.6 µmol/g of MSNs (12.5 ± 3.2 wt %) based on atomic absorption spectroscopy (AAS). In the case of AlClPc/cisplatin-MSNs, the amounts of AlClPc and cisplatin molecules loaded were 114.4 ± 25.7 µmol/g and 383.0 ± 93.3 µmol/g of material, respectively.

**Table 1:** Structural properties of MSNs, AlClPc-MSNs, cisplatin-MSNs, and AlClPc/cisplatin-MSNs.

**Sample**	Diameter (nm) *	PDI *	ζ-potential (mV) *	Diameter (nm) **	PDI **
MSNs	96.5 ± 10.5	0.19	−41.9 ± 2.0	175.5 ± 13.2	0.23
AlClPc-MSNs	107.8 ± 15.0	0.33	−45.2 ± 3.2	191.3 ± 8.6	0.33
Cisplatin-MSNs	99.1 ± 13.9	0.29	−32.1 ± 1.1	183.4 ± 7.1	0.21
AlClPc/cisplatin-MSNs	112.7 ± 19.5	0.39	−23.1 ± 2.4	198.8 ± 12.3	0.32
**Sample**	**SA (m^2^/g)**	**Pore Size (nm)**	**Pore Volume (cm^3^/g)**	**OC (%)**	
MSNs	819.7	5.3	1.57	----	
AlClPc-MSNs	712.9	5.0	1.37	7.4	
Cisplatin-MSNs	446.2	3.8	0.81	2.1	
AlClPc/cisplatin-MSNs	356.5	3.0	0.79	9.4	

* Data measured in phosphate buffer solution (1 mM; pH 7.4)/concentration of MSN = 0.1 mg/mL; ** Data measured in cell culture media (10 v % FBS)/concentration of MSN = 0.1 mg/mL PDI = Polydispersity index; SA = surface area; OC = organic content.

### 2.2. Photophysical Properties of AlClPc–MSNs, Cisplatin-MSNs, and AlClPc/Cisplatin–MSNs

**Figure 3:**
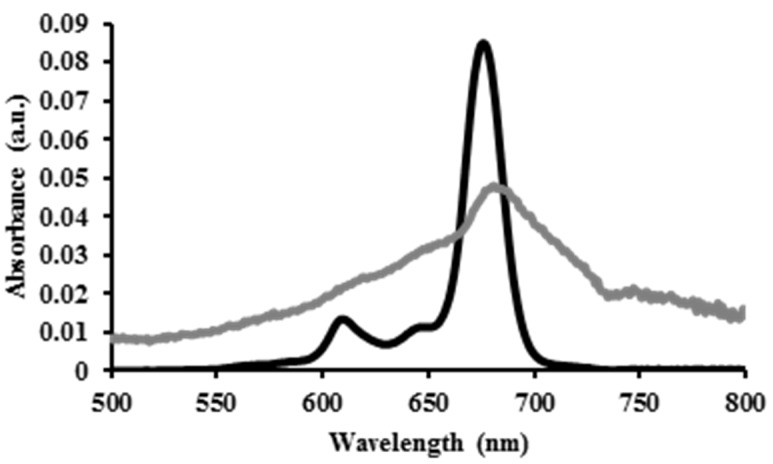
UV-VIS spectra of AlClPc molecules (**black**) and AlClPc-MSNs (**gray**).

The absorbance of AlClPc–MSN particles was determined by UV-VIS spectroscopy and compared with that of the parent PS agent. As shown in Figure 3, the absorption spectrum of AlClPc-MSNs is similar to that of AlClPc molecules. This result shows that AlClPc is robust enough to maintain similar absorption properties even after incorporation into the MSN material.

**Figure 4:**
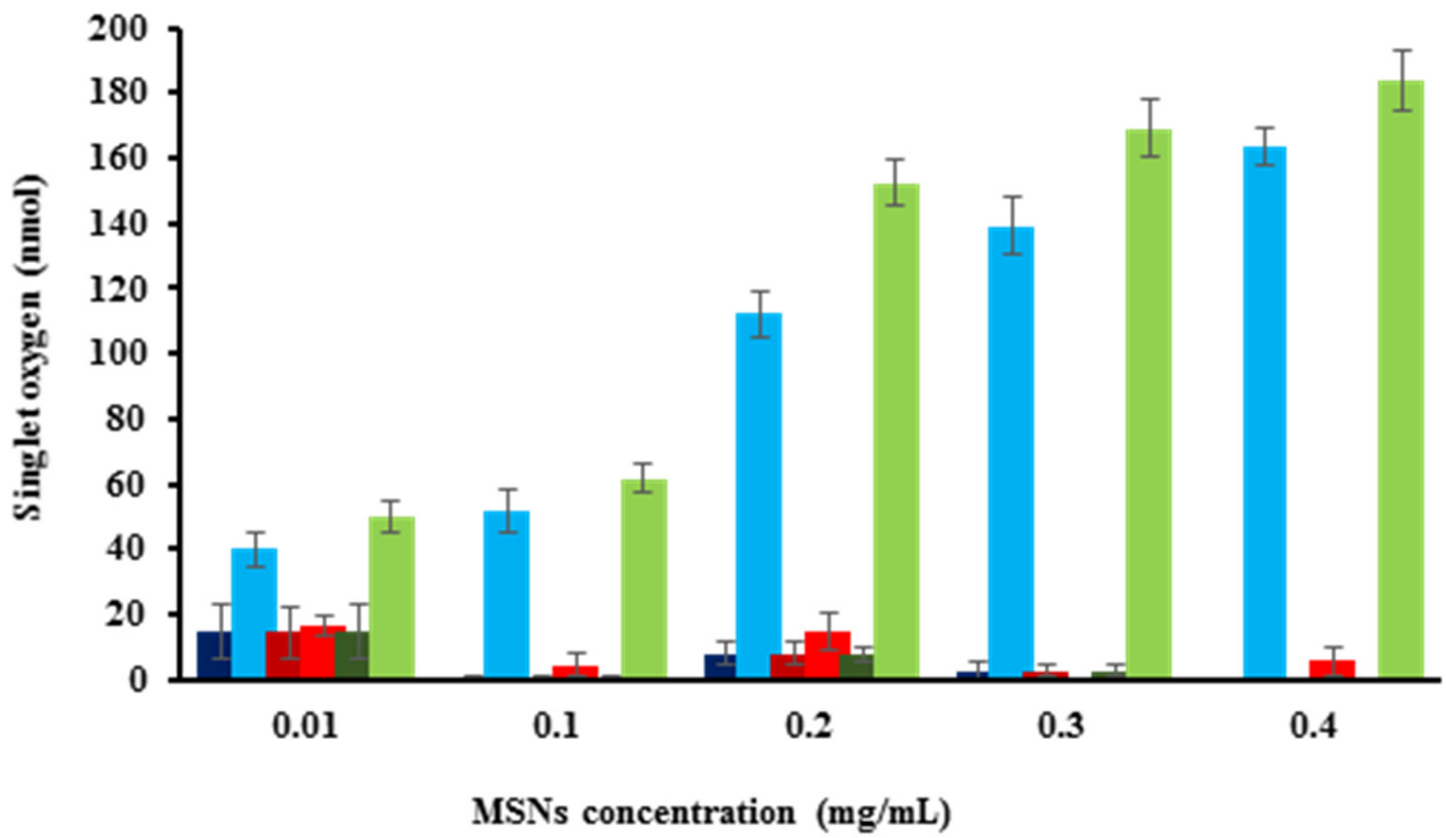
Singlet oxygen generation, without irradiation, by AlClPc-MSNs (**dark blue**), cisplatin-MSNs (**dark red**) and AlClPc/cisplatin-MSNs (**dark green**); and after irradiation, with red light, by AlClPc-MSNs (**light blue**), cisplatin-MSNs (**light red**), and AlClPc/cisplatin-MSNs (**light green**).

One of the main components of PDT is triplet molecular oxygen (^3^O_2_). Upon irradiation of light at certain wavelengths, PSs can be excited to directly transfer energy to nearby ^3^O_2_ molecules to produce ^1^O_2_. It is generally accepted that ^1^O_2_ is the principal cytotoxic component that induces significant damage to cells via apoptotic or necrotic pathways [4]. In this project, the amount of ^1^O_2_ generation in DMF solution was estimated indirectly using diphenylisobenzofuran (DPBF) as a chemical probe for ^1^O_2_. DPBF reacts irreversibly with ^1^O_2_, allowing the reaction to be easily studied by UV-VIS spectroscopy [44]. Different concentrations of MSN materials were dispersed in DMF containing DPBF. The dispersions were irradiated with red light (570–690 nm; 89 mW/cm^2^; 2.67 J/cm^2^) for 30 s, and the reduction in the absorbance of DPBF was immediately measured by a UV-VIS spectrophotometer. Figure 4 shows the ^1^O_2_ production for AlClPc-, cisplatin- and AlClPc/cisplatin-MSN materials in the absence and in the presence of red light. As expected, in the absence of red light, all of the nanoparticulate materials produced insignificant amounts of ^1^O_2_. Moreover, only the PS-loaded MSN materials generated ^1^O_2_, indicating that neither empty MSNs nor cisplatin molecules, by themselves, can activate oxygen molecules in the presence of light.

### 2.3. In Vitro Internalization, Cyto- and Phototoxicity of AlClPc–MSNs, Cisplatin-MSNs, and AlClPc/Cisplatin–MSNs

**Figure 5:**
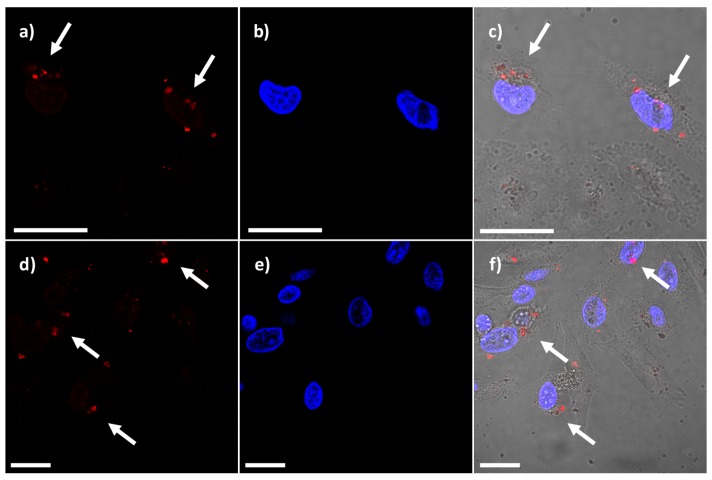
Confocal micrographs of HeLa cells incubated in the presence of 20 µg/mL of AlClPc-MSNs and AlClPc/cisplatin-MSNs for 4 h at 37 °C. (**a** and **d**) Red fluorescence from the internalized AlClPc-MSN and AlClPc/cisplatin-MSN materials; (**b** and **e**) DAPI-stained nuclei; and (**c** and **f**) overlapped image of DAPI-stained nuclei, red fluorescence, and DIC channel. White arrows indicate the localization of MSN materials. Scale bar = 20 μm.

Photosensitizer activities are not restricted solely to generation of ^1^O_2_. Many photosensitizers are bright fluorophores; some of them tend to emit in the near infrared (NIR) region, which is useful for *in vivo* imaging. Photosensitizers that also have optical imaging properties can be helpful to define and adjust parameters during PDT treatment [9]. Moreover, fluorescent PSs can aid in determining PS localization and the degree of uptake *in vitro*. In this work, we took advantage of the properties of the AlClPc molecule as a fluorescent imaging agent. Confocal laser scanning microscopy (CLSM) was used to examine the internalization of AlClPc-MSNs. HeLa cells were incubated in the presence of AlClPc-MSNs or AlClPc/cisplatin-MSNs (20 µg/mL). Red fluorescence was observed in the TRITC-channel (Figure 5a,d) due to the fluorescent imaging properties of both AlClPc- and AlClPc/cisplatin-MSN materials. As depicted in Figure 5b,e, DAPI-stained nuclei were clearly observed in the UV channel. The overlapped image between the previous micrographs and the corresponding differential interference contrast (DIC) micrograph allowed us to observed the presence of both materials within the cell bodies of HeLa cells (Figure 5c,f). Despite that DLS experiments showed that the MSN materials aggregated in cell culture media (Table 1), the results from confocal microscopy strongly indicate that AlClPc- and AlClPc/cisplatin-MSNs were uptaken by HeLa cells. Previous reports have shown that HeLa cells can handle the internalization of cubic particles as large as 2–3 µm, presumably through the combination of energy-dependent phagocytosis and a clathrin-mediated mechanism [45]. In particular, Nel and co-workers have shown that MSNs with different aspect ratio and hydrodynamic diameters above 200 nm in cell culture media can be internalized by HeLa cells by a macropynocytosis process [43]. Additional experiments, which are out of the scope of this work, are neccessary to investigate whether the MSN materials fabricated in this study are uptaken by macropynocitic and/or endocytic pathways.

**Figure 6:**
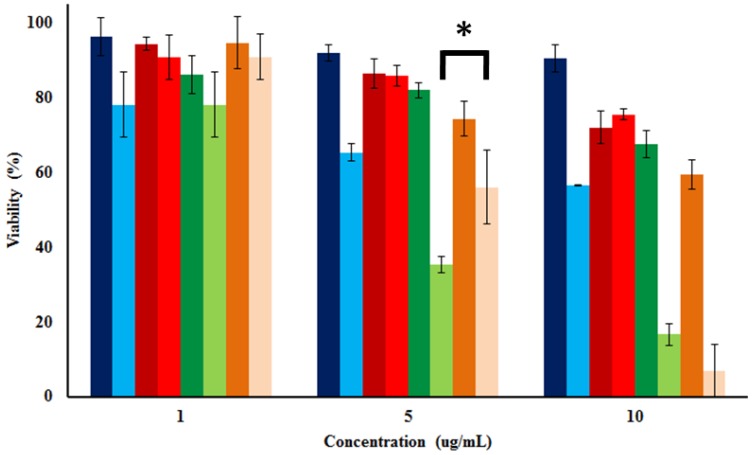
Cytotoxicity of AlClPc-MSNs (**dark blue**), cisplatin-MSNs (**dark red**), AlClPc/cisplatin-MSNs (**dark green**) and physical mixture of cisplatin/AlClPc molecules (**dark orange**) under dark conditions. Phototoxicity of AlClPc-MSNs (**light blue**), cisplatin-MSNs (**light red**), AlClPc/cisplatin-MSNs (**light green**) and physical mixture of cisplatin/AlClPc molecules (**light orange**) after red light exposure (570–690 nm; 89 mW/cm^2^) for 20 min. Asterisk indicates *p* < 0.05.

The toxicity of different concentrations (1, 5, and 10 µg/mL) of AlClPc-, cisplatin-, AlClPc/cisplatin-MSN materials, and the equivalent amounts of a physical mixture of cisplatin/AlClPc (1 µg/mL = 0.384/0.114 µM; 5 µg/mL = 1.92/0.57 µM; and 10 µg/mL = 3.84/1.14 µM) was tested under red light exposure (570–690 nm; 89 mW/cm^2^; 106.8 J/cm^2^) for 20 min. Figure 6 shows the survival numbers of HeLa cells that were incubated for 24 h in the presence of the materials and drugs, both without irradiation (dark toxicity) and after light irradiation. We did not observe significant differences in the toxicity of the MSN particles at lower concentrations (1 µg/mL) or the equivalent mixture of cisplatin/AlClPc molecules, regardless of whether or not the samples were irradiated with red light. After increasing the concentration of MSN materials to 5 and 10 µg/mL, though, we observed major differences in the toxicity results. AlClPc-MSNs did not significantly reduce the viability of HeLa cells under dark conditions, but after irradiating the nanoparticles with red light, the viability of cells decreased approximately 35.0% and 45.0%. These data indicate that AlClPc-MSN particles generate ^1^O_2_, after irradiation with light, resulting in phototoxic effects for HeLa cells. The cytotoxicity of cisplatin-MSNs, meanwhile, is concentration dependent but is not affected by irradiation with light. The viability of HeLa cells in the presence of 5 and 10 µg/mL of the cisplatin-loaded particles was reduced by approximately 15.5% and 26.5%. Based on the quantity of cisplatin loaded, and on the concentration of cisplatin-MSNs in cell media, the calculated concentration of released cisplatin molecules is around 4.2 µM. The IC_50_ of cisplatin, for HeLa cells under similar conditions, is 17.9 ± 4.5 µM (data not shown). Therefore, it is not expected that cisplatin-MSNs reduce the viability of HeLa cells by more than 50%. In the case of the AlClPc/cisplatin-MSNs, we observed that the cytotoxicity under dark conditions is similar to that of the cisplatin-MSNs, as both materials contain similar amounts of cisplatin (12.5 ± 3.2 wt % *versus* 11.5 ± 2.8 wt %). In the same way, the physical mixture of drugs did not show statistically significant difference in the cell survival in comparison with the AlClPc/cisplatin-MSNs in the absence of light. By contrast, however, the viability of HeLa cells in the presence of 5 and 10 µg/mL of the AlClPc/cisplatin-loaded particles was reduced by approximately 65.0% to 85.0%, respectively, after irradiation. Interestingly, the AlClPc/cisplatin-MSNs (5 µg/mL) showed a statistically significant difference in the reduction of the cell survival in comparison with the equivalent amount of the physical mixture of cisplatin/AlClPc, but not difference was observed at 10 µg/mL. To find out whether there is just an additive effect of the MSN materials for the decrease of cell survival, we added the reduction in cell viability upon light irradiation for the individual AlClPc- and cisplatin-MSN materials, at each concentration, to estimate a total decrease in HeLa viability of 50.5% for 5 µg/mL and 71.5% for 10 µg/mL. Hence, we observed a slight increase in the toxicity from the MSN platform simultaneously loaded with both compounds. In addition, AlClPc/cisplatin-MSNs have similar or improved phototoxic effect than the physical mixture of drugs. We conclude that the concurrent transport and delivery of both the AlClPc photosensitizer and of the cisplatin drug from MSNs potentiate their toxicity against HeLa cells [46]. Additional experiments need to be done to determine whether this is a synergistic effect or not. Nevertheless, these results show the potential of using MSNs as nanocarriers for a combination therapy (photo- and chemotherapy) to treat cancer.

## 3. Experimental Section

### 3.1. Materials and Methods

All of the reagents were purchased from Aldrich and used without further purification. Thermogravimetric analysis (TGA) was carried out using a Mettler Toledo TGA/SDTA851 instrument (Mettler-Toledo AG Analytical, Schwersenbach, Switzerland) with a platinum pan and a heating rate of 5 °C/min under a nitrogen atmosphere. A Raith 150 field emission scanning electron microscope (SEM) (Raith America Inc., New York, NY, USA) was used to determine particle size and shape. Nanoparticle samples were suspended in ethanol in preparation for the SEM. A drop of the suspension was placed on a silicon wafer and the solvent was allowed to evaporate. A JEOL JEM 2100 LaB6 transmission electron microscope (TEM) (JEOL USA, Inc., Peabody, MD, USA) was used to corroborate particle size and morphology. Each TEM sample was prepared by suspending the nanoparticles in methanol. A drop of the suspension was placed on a TEM carbon grid (200 mesh) and the solvent was allowed to evaporate overnight. Dynamic light scattering (DLS) and ζ-potential measurements were carried out using a Malvern Instrument Zetasizer Nano (Malvern Instrument Ltd., Malvern, UK). The number of AlClPc and of cisplatin molecules loaded into the MSNs was quantified by UV-VIS spectroscopy (Cary 300 Bio UV/VIS spectrometer, Varian, Sidney, Australia) and atomic absorption spectroscopy (PerkinElmer Analyst 200 equipped with a graphite furnace HGA 900 (PerkinElmer, Waltham, MA, USA)).

### 3.2. Synthesis of MSN Materials

MSNs were synthesized using a surfactant-templated method from the literature, with slight modifications [42]. Briefly, 0.2 g of CTAB, along with 96.0 mL of water, 0.7 mL of sodium hydroxide (2 M solution), and 1.4 mL of mesitylene, were mixed together. This solution was heated to 80 °C for 45 min at 800 rpm. To this solution, 1.0 mL of tetraethylorthosilicate (TEOS) was rapidly added while the solution was stirred at 950 rpm. The reaction was stirred for an additional 2 h at 80 °C. The as-made MSNs were isolated by centrifugation at 13,000 rpm over 8 min. MSNs were washed with water and ethanol three times and then redispersed in ethanol. The CTAB was extracted by an acidic wash procedure after the nanoparticles were made. For example, 350 mg of as-made MSNs were redispersed in 175 mL of an acidic solution of methanol (HCl (37%), 1.0 mM). The dispersion was stirred overnight at 60 °C. The material was then washed with water twice and ethanol before being stored in ethanol.

To load the AlClPc compound into MSNs, 150 mg of the nanoparticulate material were redispersed in 50 mL of a DMSO solution of AlClPc (1.0 mM). The dispersion was stirred for 24 h at room temperature. The AlClPc-MSNs were separated by centrifugation at 13,000 rpm for 5 min. The final material was washed with DMSO at least two more times and stored in ethanol. The supernatant and washing solutions were collected to determine the amount of AlClPc loaded to MSNs by calculating the difference with the initial amount of AlClPc. In a similar way, cisplatin was loaded into MSNs by stirring 50 mg of nanoparticles in 15 mL of a DMSO solution of cisplatin (3.0 mM) for 24 h at room temperature. The final material was washed with DMSO at least two more times and stored in ethanol. The supernatant and washing solutions were collected to determine the amount of cisplatin loaded into the MSNs by calculating the difference from the initial amount of cisplatin. To fabricate the AlClPc/cisplatin-MSN material, 50 mg of the previously synthesized AlClPc-MSNs were stirred in 15 mL of a DMSO solution of cisplatin (5.0 mM) for 48 h at room temperature. The final material was washed with DMSO at least two more times and stored in ethanol. The supernatant and washing solutions were collected to determine the amount of AlClPc and cisplatin loaded to MSNs by calculating the difference from the initial amount of the compounds.

### 3.3. Characterization of the Photophysical Properties of MSN Materials

A Cary 300 Bio UV/VIS spectrometer was used to determine the absorption of the AlClPc-MSNs and of the AlClPc/cisplatin-MSNs in DMSO.

The generation of singlet oxygen was determined indirectly by using a ^1^O_2_ probe, 1,3-diphenylisobenzofuran (DBPF) [44] .The samples were immediately prepared by transferring 40 µL of DPBF in dimethylformamide (DMF) stock solution (8 mM) to 4 mL of a DMF suspension of AlClPc-MSNs, cisplatin-MSNs, or AlClPc/cisplatin-MSNs. Different concentrations (0.01, 0.1, 0.2, 0.3, and 0.4 mg/mL) of MSN materials were used for this assay. The experiments were carried out by irradiating the samples with a LumaCare LC122 light source (FOP LUM CL01, 570–690 nm; 89 mW/cm^2^) for 30 s. The decrease of DPBF absorbance at 415 nm was monitored with a Cary 300 Bio UV/VIS spectrometer. The amount of ^1^O_2_ produced was calculated by considering the difference between the initial amount and the final amount of DPBF after red light exposure.

### 3.4. In Vitro Internalization of MSN Materials

For *in vitro* experiments, the MSN materials are centrifuged down, the ethanolic solution is disposed, and the particles are redispersed in cell culture media for inoculation. HeLa cells were seeded at a density of 1 × 10^5^ cells per mL in six-well culture plates, with coverslips at the bottom of the wells, and incubated in 3 mL of RPMI-1640 cell media for 24 h at 37 °C with 5% CO_2_. The cell media was replaced by 3 mL of AlClPc- and AlClPc/cisplatin-MSN materials (20 µg/mL) dispersed in the RPMI-1640 cell media and the cells were incubated for 4 h in this dispersion. Finally, for each independent experiment, the cell-plated coverslips were washed twice with PBS buffer (1 mM, pH 7.4) and stained with NucBlue® Live cell staining DAPI solution for 15 min. The stained coverslips were placed in microscope slides and examined under an Olympus Fluoview FV 1000 (Olympus America Inc., Center Valle, PA, USA) confocal fluorescence microscope system.

### 3.5. Cyto- and Photo-Toxicity of MSN Materials

HeLa cells were seeded at a density of 1 × 10^4^ cells per mL in a 96-well cell plates and incubated in 100 µL of RPMI-1640 cell media for 24 h at 37 °C with 5% CO_2_. Cells were then inoculated with AlClPc-MSNs, cisplatin-MSNs or AlClPc/cisplatin-MSNs (1, 5, and 10 µg/mL) or the equivalent amount of drugs for the physical mixture of cisplatin/AlClPc (0.384/0.114, 1.92/0.57, and 3.84/1.14 µM) for 24 h in cell media, followed by PBS washing steps, and then further incubated in PBS for light exposure. Samples were exposed to a LumaCare LC122 light source (FOP LUM CL01, 570–690 nm; 89 mW/cm^2^, MBG Technologies Inc., Newport Beach, CA, USA) for 20 min. The light source has a homogeneous illumination in an area of 25 cm^2^. After irradiation, the cells were incubated in cell media for another 24 h and the cell survival was tested by the MTS assay. The absorbance was measured at a wavelength of 450 nm. Cell viability percentage was calculated based on the absorbance measured relative to that of control culture cells. The cytotoxicity under “dark conditions” was determined following the same protocol, but without light exposure. To rule out any possibility of cytotoxicity due to the use of DMSO for drug loading, MSNs were stirred in the presence of DMSO for 48 h, washed twice with DMSO and stored in ethanol. Cell viability was determined by MTS following the protocol described above (Figure S3).

## 4. Conclusions

This work explores the synthesis and characterization of a MSN-based platform to simultaneously carry photosensitizer molecules and anticancer drugs for combination therapy. The structural and photophysical properties of the materials were characterized by different techniques. DLS and ζ-potential measurements showed that the MSN materials aggregate in simulated physiological conditions (PBS, 1 mM, pH = 7.4) and that their surfaces are negatively charged due mainly to the presence of siloxides. The surface area, pore size, and volume values calculated by the BET and BJH methods, using the N_2_ sorption isotherms, corroborated that the MSNs synthesized in this work have high surface areas, large pore sizes, and large volumes. Moreover, after the dual loading of AlClPc and cisplatin molecules, the observed decrease in these values indicates that these compounds have been successfully loaded into the MSNs. SEM and TEM corroborated the synthesis of MSNs with an average particle diameter of 79.7 ± 13.3 nm. The incorporation of AlClPc molecules into MSNs does not affect the light absorption properties of this photosensitizer. The singlet oxygen generation assay demonstrated that only MSN particles containing the photosensitizer AlClPc can produce ^1^O_2_ after exposure to red light. The *in vitro* confocal microscopy experiments showed that the MSN materials can be readily internalized in HeLa cells. To carry out this experiment, we used the photophysical properties of the AlClPc molecule as a fluorescent imaging agent. Finally, the cytotoxic experiments, performed after light irradiation, demonstrated that the combination of both AlClPc and cisplatin compounds in one MSN platform potentiate the toxic effect against HeLa cells in comparison to the individual AlClPc-MSN, cisplatin-MSN materials and the physical mixture of both drugs. This combinatorial MSN system still needs to be evaluated in cisplatin-resistant cells; nevertheless, our current results validate the potential of using MSNs as nanocarriers for combination therapy (photo and chemotherapy) to treat cancer. Moreover, this MSN platform can be further functionalized with polymers and targeting agents to render colloidal stability and enhance the accumulation of the material in cancer cells.

## Supplementary Materials

Supplementary materials can be accessed at: http://www.mdpi.com/2079-4991/5/4/2302/s1.
