# Supplementary material for: Mesoporous Silica Nanoparticles Loaded with Cisplatin and Phthalocyanine for Combination Chemotherapy and Photodynamic Therapy in vitro
Source: Nanomaterials (Basel). 2015 Dec 16;5(4):2302–16. doi: 10.3390/nano5042302 (PMC5304775; doi:10.3390/nano5042302)
Supplement: Supplementary file 1 [file nanomaterials-05-02302-s001.pdf]

Supplementary Information

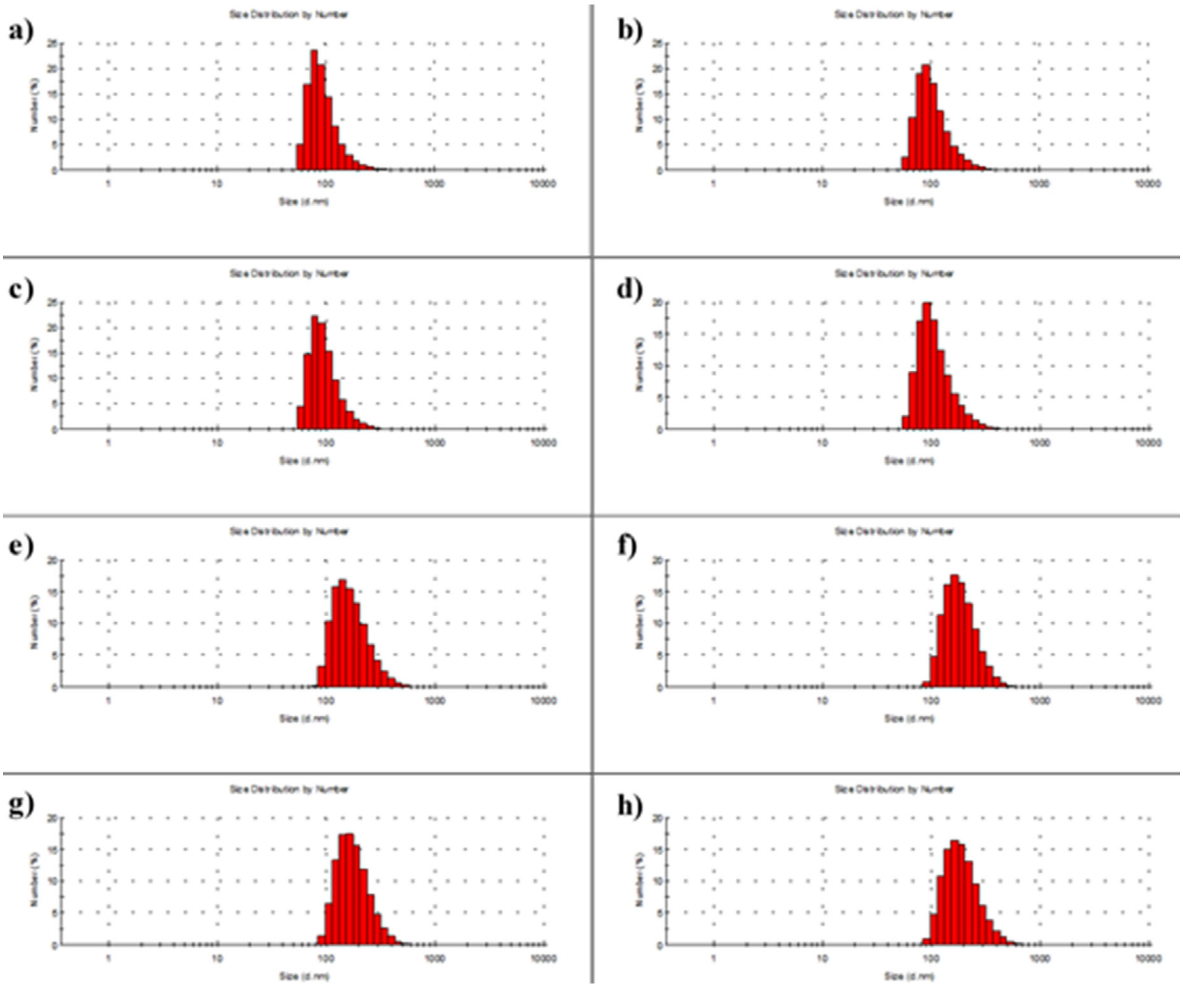

Figure S1. Particle size distribution histogram of (**a,e**) MSNs, (**b,f**) AlClPc-MSNs, (**c,g**) cisplatin-MSNs and (**d,h**) AlClPc/cisplatin-MSNs in 1.0 mM phosphate buffer solution (**a–d**) or cell culture media (10 v % FBS) (**e–h**).

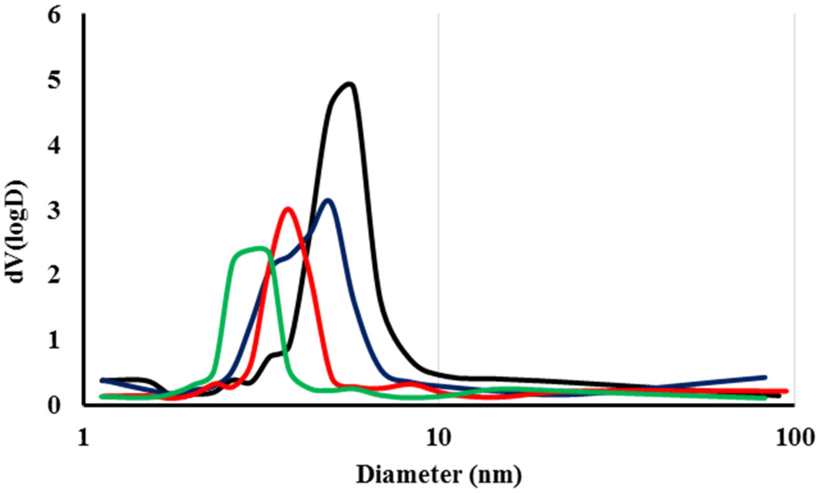

Figure S2. Pore size distribution of MSNs (**black**), AlClPc-MSNs (**blue**), cisplatin-MSNs (**red**) and AlClPc/cisplatin-MSNs (**green**).

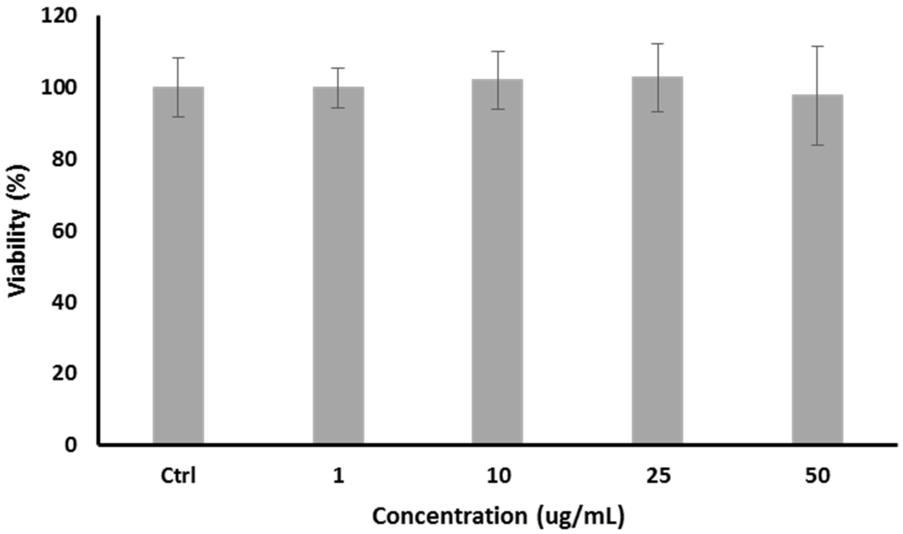

Figure S3. Cytotoxicity of MSNs stirred in the presence of DMSO for 48 h, washed twice with DMSO and stored in ethanol.

© 2015 by the authors; licensee MDPI, Basel, Switzerland. This article is an open access article distributed under the terms and conditions of the Creative Commons Attribution license (http://creativecommons.org/licenses/by/4.0/).
